# Characteristics of transplant athletes competing at national and international transplant games

**DOI:** 10.1136/bmjsem-2021-001248

**Published:** 2022-02-28

**Authors:** Thomas Hames, Sheila Leddington-Wright, Charles Douglas Thake, Mike Price

**Affiliations:** Health and Life Sciences, Coventry University, Coventry, UK

**Keywords:** assessing physical training modalities in enhancing sports performance, athlete, exercise physiology, training, sports

## Abstract

**Objective:**

To describe the characteristics of athletes with solid-organ transplants (TxA) attending the British and World Transplant Games.

**Methods:**

220 TxA completed an online survey to explore transplant history, medications, training advice and support and limitations to training.

**Results:**

TxA were predominantly caucasian, male, kidney recipients in their mid-forties and approximately 11 years post-transplant. The majority of TxA took some form of medication (immunosuppressants 88%, steroids 47%, antihypertensives 47%, statins 28%, antiplatelets 26%, antibiotics/antivirals/antifungals 20%). Stem cell recipients were least likely to require medication. Post-transplant complications were experienced by 40% of TxA, with 53% of these being rejection. Although over half the participants (57%) initially received exercise or training advice post-transplant, only 34% of these received this from their consultants or immediate medical team. Only 1% had been specifically directed towards transplant sport. Half of the TxA (53%) perceived there were limitations preventing them from performing at their potential, 45% considered they did not recover from training as well as non-TxA while 29% felt they trained equally to non-Tx’s. Only 6% considered medication impaired training. TxA competed for a range of reasons from social and health benefits to winning medals.

**Conclusions:**

TxA compete at the British and World Transplant Games for a diverse range of reasons. Athletes manage a range of medications with a range of exercise and health experiences pre-transplant. TxA face a lack of both general and specific exercise training and recovery guidance. The individuality of each TxA‘s background should be considered and is likely reflected in their exercise capacity and goals.

**Key messages**

**What is already known on this topic**

- Athletes with solid-organ transplants (TxA) demonstrate reduced physiological responses to exercise.
- Reduced peak oxygen uptake results from chronic reductions in muscle mass and muscle quality prior to surgery.
- Post-transplant, physiological responses may not recover due to immunosuppressant drug interactions.
- Competitors in transplant events are reported to be predominantly male kidney recipients between 42 and 46 years of age, managing comorbidities and concurrent medications. The characteristics and needs of TxA has received little attention.

**What this study adds**

- The majority of TxA manage multiple medications, often being specific to their transplant type.
- Many competitors attend the Games for the health-related benefits and fitness, competing for enjoyment, with a smaller proportion competing to win.
- Few competitors follow any structured training advice from exercise professionals.
- Each TxA’s journey differs with respect to transplant type and exercise experience which is likely reflected in their exercise capacity and goals.

**How this study might affect research, practice or policy**

- There is a need to understand current TxA training practices and develop appropriate high performance, sport-specific training guidance post-transplant for those recipients wishing to compete.
- Practitioners should be aware that a TxA desire to compete is multifaceted and often beyond that of athletic competition alone. Each competitor has a unique background to consider.

## Introduction

Determining the characteristics of athletic populations enables optimisation of training and performance.^1–3^ For coaches and other members of the support team in daily contact with athletes, knowledge of their specific requirements is essential.^4^ Some athlete groups though (eg, asthma, diabetes, disability) present the support team with an additional challenge of managing medical conditions and medication around daily training and competitions. A group of athletes facing considerable medication challenges are athletes with solid-organ transplants. Transplant recipients also demonstrate reduced physiological responses to exercise, such as peak oxygen uptake (VO_2peak_). Post-transplant, reduced VO_2peak_ may be a result of chronotropic incompetence in an otherwise well-functioning allograft, such as for heart recipients or allograft dysfunction. However, key contributors to reduced VO_2peak_ are chronic reductions in muscle mass and muscle quality prior to surgery that may not recover post-transplant due to immunosuppressant drug interactions.^5^ Although position stands relating to athletes with asthma^6^ and diabetes^7^ exist, the characteristics and needs of athletes with solid-organ transplants (TxA) has received little attention.

Since the inaugural Transplant Games in 1978 participant numbers have grown from 99 competitors representing five countries to 1500 competitors representing 69 countries at the 2017 World Transplant Games in Malaga.^8^ Despite this increase in participation, there is limited empirical data regarding demographics, physical characteristics and medication regimes of TxA attending the Games. Existing data suggests participants at Transplant Games have predominantly been male kidney recipients with a mean age of 42–46 years^9–13^ Data from the 1996 US Transplant Games^9^ and 2012 Latin American Transplant Games^13^ reported a high proportion (52%) of competitors managing one or more comorbid conditions while a small proportion (7%) experienced graft rejection within the 12 months prior to competition. Competitors at these events managed concurrent medications with continuous immunosuppressant management evident among nearly all TxA. In addition to medications, TxA differ from other athlete groups as they compete with respect to age across a range of events. As such, typical training guidelines are unlikely able to cater for the wider age range of TxA’s. Furthermore, the specific journey of each transplant recipient differs considerably.^14^ To our knowledge, no study examining TxA has concurrently reported the training advice received, perceptions of training and performance limitations alongside generic transplant information which has the potential to support both transplant recipients and practitioners presurgery and postsurgery. The aim of this study was to determine the characteristics of TxA attending the British and World Transplant Games.

## Method

### Participants

A survey-based study was completed by 220 English speaking TxA’s (male: 139, female: 81). Gatekeeper consent for national and international standard athletes was provided from the World Transplant Games Federation, Transplant Sport UK and national team managers as part of the institutional ethics process. The questionnaire was advertised on the World Transplant Games Federation and Transplant Sport UK websites. The survey was open for ~10 weeks from 12 June 2017 to 1 September 2017. No reminders to complete the survey were posted. Participants provided individual consent which was built into the questionnaire. Inclusion criterion was successful entry into the 2017 British or World Transplant Games which, in doing so, confirmed the TxA had met the required conditions for competition, that is, having received one or more life supporting allografts (kidney, liver, heart, stem cell, lung, pancreas, intestine), be more than 6 months post-transplant and with a stable allograft and medically fit as signed off by their doctor.^8^

### Survey procedure

A 63-question survey was developed to explore training practices, recovery postexercise, training advice and support undertaken and experienced by TxA and is fully available as online supplemental appendix 1. Questions relating to TxA characteristics (sex, age, height, body mass (enabling body mass index calculation; BMI, mass/height^2^; kg/m^2^), nationality and ethnicity), transplant history (initial reason for transplant and age at transplant), medications, complications, source of exercise/training advice and reasons for attending Tx Games are reported here.

10.1136/bmjsem-2021-001248.supp1 Supplementary data

Participants completed the survey on the Bristol Online Survey platform using a web link available through the World Transplant Games Federation web page and consenting national team web pages. Further recruitment occurred through word of mouth at both the British Transplant Games (Glasgow; 27 July 2017–30 July 2017) and the World Transplant Games (Malaga; 24 June 2017–30 June 2017). The survey was piloted using transplant athletes (n=4) to assess clarity and practicality of completion. Feedback from the pilot study indicated the survey allowed participants to reflect on their journey from illness to athlete and what they have been able to achieve through physical training. Furthermore, participants indicated they were happy to answer all questions, taking between 15 and 20 min to complete.

### Statistical analysis

Survey analysis was predominantly descriptive in nature with frequency distributions for the whole group and for males and females, type of transplant, etc as appropriate reported. Normality was determined using the Kolmogorov-Smirnov test prior to independent t-tests and two-way analysis of variance for the comparisons between subgroups for sex, age, height, body mass and BMI.

### Patient and public involvement

Althetes with solid organ transplants were involved in piloting the questionnaireand will be involved in the dissemination plans of this research.

## Results

### Participant characteristics

The overall ratio of male:female TxA was 63:37%. Within the five largest subgroups based on transplant type liver recipients had fewer males whereas stem cell and lung recipients had more males (table 1). With the exception of lung recipients, where males and females were of similar mass (p=0.686), males were generally heavier than females (p=0.006). BMI was comparable across the five largest sub-groups. Mean age of TxA was similar across the majority of subgroups with similarly large age ranges across groups. Time since transplant was also similar across transplant type. The majority of participants (57%) acquired a chronic illness in adulthood resulting in organ transplantation with smaller frequencies for those undergoing transplant as a child (19%), children reaching adulthood prior to transplant (16%) and adults with a sudden onset of illness leading to transplant (9%). Athletes were predominantly white caucasian when considered as the whole group, UK and non-UK countries (90, 95, 83%, respectively). All other ethnic groups represented less than 5% of both UK and non-UK participants.

**Table 1:** Characteristics of competitors attending the British and World Transplant Games (n=220)

			Transplant type								
			All	Kidney	Liver	Heart	Stem cell	Lung	Heart & Lung	Kidney & Pancreas	Pancreas
N			220	95	50	35	21	11	4	3	1
Sex	Male	n	139	61	27	23	16	9	1	2	–
	Female	n	81	34	23	12	5	2	3	1	1
Age (years)	All	Mean (±SD)	45±15	42±15	47±17	48±13	51±17	42±16	40±7	40±3	53
		Range	77	63	63	50	75	52	18	6	–
	Male	Mean (±SD)	45±16	43±15	49±20	45±13	52±18	43±18	39	39±4	–
		Range	77	63	63	49	75	52	0	6	–
	Female	Mean (±SD)	44±14	42±15	44±13	52±12	48±15	38±4	41±9	42	53
		Range	53	53	40	41	33	15	18	–	–
Mass (kg)	All	Mean (±SD)	73±16	73±15	73±16	76±17	75±16	68±13	59±11	88±22	52
		Range	103	102	71	63	52	48	24	43	–
	Male	Mean (±SD)	78±15	78±14	80±16	82±15	79±16	68±15	57	99±13	–
		Range	97	93	65	56	52	48	–	18	–
	Female	Mean (±SD)	64±12	64±14	65±11	65±15	62±5	65±6	60±13	65	52
		Range	63	62	40	46	13	8	24	–	–
Height (cm)	All	Mean (±SD)	172±11	171±10	172±11	176±10	177±12	173±6	162±7	165±12	158
		Range	54	50	56	35	45	20	16	23	–
	Male	Mean (±SD)	177±9	175±8	178±10	181±7	181±10	174±6	151	172±3	–
		Range	55	38	48	23	31	18	–	4	–
	Female	Mean (±SD)	164±8	164±10	165±7	166±8	164±8	169±5	165±2	151	158
		Range	35	41	25	27	18	7	4	–	–
BMI (kg.m2)	All	Mean (±SD)	24.5±4.1	24.9±4.4	24.6±4.4	24.3±4.3	23.4±2.5	22.3±3.5	22.8±3.9	32±3.6	21
		Range	25	24	18	16	9	12	8	7	–
	Male	Mean (±SD)	24.9±3.9	25.6±3.8	25.1±4.5	24.9±3.5	23.5±2.9	22.3±3.9	25	33.5±3.5	–
		Range	23	22	16	11	9	12	–	5	–
	Female	Mean (±SD)	23.7±4.0	23.8±3.8	24±4.2	23±5.8	23±1	22.5±0.7	22±4.4	29	21
		Range	19	18	18	16	2	1	8	–	–

Grouped according to transplant subgroup, as per British Transplant Games and World Transplant Games competitions.

BMI, body mass index.

### Medications and complications

TxA took an average of 5±3 medications each week and were advised to take daily vitamins or minerals (see online supplemental information). The greatest number of weekly medications was reported by lung recipients (8±4), whereas stem cell recipients managed the least (2±2), 43% (n=9) of whom took no medication. One or more immunosuppressants were manged by 88% of TxA, of which, the most frequent users were heart recipients (100%), while stem cell recipients were least frequent users (10%). Steroid medication was managed by 47% of TxA of which lung recipients managed the most (82%) and liver recipients managed the least (26%). Antihypertensive management (47%) was apparent in all Tx subgroups, heart recipients managed the most (69%) whereas stem cell recipients managed the least (10%). Statin and antiplatelet medications were managed by 28% and 26% of TxA, respectively, predominantly by heart recipients (80%, 37%, respectively) and least frequently by stem cell recipients (0%, 5%, respectively), with no lung recipients taking antiplatelets. Antibiotics/antiviral/antifungal medication use was reported in 20% of TxA and was observed across all Tx subgroups (eg, lung recipients 90%, kidney recipients 9%). Complications post-Tx were experienced by 40% (n=88) of TxA (table 2). Organ rejection whether minor or complete accounted for 53% of all complications and, while prevalent within all Tx subgroups, rejection complications were greatest among heart recipients accounting for 67% of all complications. Infection and associated organ damage were both experienced by 10% of TxA experiencing complications. Graft versus host disease was experienced among 50% of stem cell recipients.

10.1136/bmjsem-2021-001248.supp2 Supplementary data

**Table 2:** Prevalence of complications within competitive transplant recipients and the five most common subgroups since transplant

	Total	Kidney	Liver	Heart	Stem cell	Lung
No of complications (Percentage within transplant group)	88/220 40	37/95 39	23/50 46	12/35 34	8/21 38	7/11 64
Rejection, minor rejection (%)	53	51	65	67	13	43
Cancer/lymphoproliferative disease (%)	6	3	4	–	13	29
Other organ damage (%)	10	16	9	–	13	14
Anaemia (%)	2	3	–	8	–	–
Infection (%)	10	11	9	17	–	14
Graft versus host disease (%)	5	–	–	–	50	–
Other (%)	13	16	13	8	13	–

### Exercise advice

Post-transplant 57% of TxA received training guidance or advice from sources such as; consultant/medical support team (34%), supporting therapists (8%), coach (4%), family and friends (4%), another recipient (2%) or other (5%). When given, training advice consisted of; encouraging physical activity and given some form of criteria (25%), directed towards therapy classes (23%), told to take it gentle and slow but nonspecific (8%). A small proportion of TxA were unclear on what had been advised (3%), whereas only 1% were encouraged to explore the Tx Games.

### Performance limitations

Over half the participants (53%) perceived that there was an existing limitation preventing them from performing at their true potential. Of these, current injury or illness (23%), lack of fitness/strength (18%), fear of overdoing it and lack of motivation (13%), finance and time limitations (10%), medication (8%), a lack of understanding from coaches and supporting networks (8%) were reported. However, 43% of participants (n=96) were actively trying to improve on these factors by increasing levels of activity (37%), seeking further advice from Sports Therapists and Physiotherapists (13%) and factoring in more time to train (6%). Some TxA though believed nothing would change in relation to their current limitations (17%).

### Perceived training intensity

When asked to compare their ability to train to that of an event matched non-TxA 29% felt they trained equally, 21% felt they could train the same for 75% of the time, 19% could train the same 50% of the time, whereas 16% reduced the intensity of their training session from the start of the session. The greatest training adjustments were reported by heart recipients with 37% modifying their sessions from the start and 23% adjusting up to 50% of a training session. When considering the recovery between sessions on back-to-back training day’s 55% of all TxA believed they recovered equally to an event matched non-TxA whereas over half (52%) of heart recipients believed their recovery was impaired. For the whole group, 14% believed chronic fatigue impaired recovery, being greatest for the heart-recipients (26%) and least for the lung recipients (9%). Although only 6% of the whole group believed medication impeded their recovery, this was greatest among heart recipients (14%) with no stem cell or lung recipients reporting this.

### Participation reasons

When exploring reasons why TxA participated at various Transplant Games 65% wanted to improve their fitness, 62% attended to be part of the Tx community, 61% competed for fun, 60% participated to encourage a healthier lifestyle, 31% aimed to win international events, 27% aimed to win national events, 20% attended to compete at events they competed in prior to transplant, 16% to compete at new events and 12% attended to break records.

## Discussion

To our knowledge, this is the first study to report the characteristics of TxA’s attending the British and World Transplant Games. The main findings were that TxA were generally caucasian, male, kidney recipients in their mid-forties approximately 11 years post-transplant. Stem cell recipients were potentially the only TxA not commonly requiring medication, otherwise, all TxA took some form of medication. A high proportion of all TxA experienced complications post-transplant. Just over half the participants had received guidance or advice on exercise or training post-transplant, with only 1% being directed towards the Transplant Games or Transplant Sport. A high proportion of TxA perceived there were limitations preventing them from performing at their potential, yet 29% felt they trained equally to non-TxA’s. The majority participated at the Transplant Games to improve their fitness and be part of the transplant community. Only a small proportion attended to win titles and break records.

### Participant characteristics

The age, male:female ratio and BMI of the current study are similar to previous reports of TxA.^9 10^ Competitors are generally older than expected for non-transplant athletes at international competitions (ie, ~23–28 years)^15 16^ as a large proportion of the TxA received their transplant during adulthood subsequent to chronic illness as a child. Furthermore, transplant sport involves competing in age categories up to 70+years,^8^ thus, many older competitors exist than would be expected for non-transplant sport.

### Medications and complications

While medications play a vital role in maintaining a healthy lifestyle and transplant homeostasis, they also carry associated risks. For example, immunosuppressants (eg, tacrolimus or ciclosporin) can increase the risk of infections, for which approximately 20% of TxA in the current study were prescribed antibiotics. Long-term side effects of immunosuppressants include kidney disease, high blood pressure and cholesterol, diabetes, osteoporosis and the risk of certain cancers,^17^ which may require further medical intervention. Steroids (eg, prednisone) also carry potential side effects including hypertension, hypercholesterolaemia, high blood glucose, weight gain and anxiety.^12^ In comparison to other transplant types, stem cell recipients took the fewest medications. Practitioners working with TxA should be aware of the specific type of transplant and the likely medications taken as well as their effects on exercise—as has been noted for commonly prescribed medications.^18^ However, the specific effects of typical transplant related medications on exercise responses are generally unreported as the focus of many such medications are, understandably, to offset allograft rejection. Physical activity and exercise though can play a pivotal role in negating common side effects improving health-related quality of life compared with sedentary transplant recipients^19 20^ and comparable to the general population.^21^ Indeed, BMI of the current participants was below values associated with graft dysfunction and poor survival (ie, <25 kg/m^2^,^22^ <30 kg/m^2^ 23). Complications though were experienced by the TxA being predominantly minor or major allograft rejection, which remains the greatest barrier to successful transplantation.^24^ Previous research of TxA at the 1996 US Transplant Games reported a greater prevalence of TxA competing with one or more underlying comorbid conditions than this study (79% vs 40%, respectively).^9^ Thus, managing complications or comorbid conditions post-Tx is not regarded as uncommon, for which exercise practitioners should be aware.

### Exercise advice

Although it was encouraging that 57% of TxA were given exercise and training advice post-transplant this generally referred to low intensity exercise for health and ‘not to overdo it’. Plausible reasons are most likely related to post-transplant immunosuppressant doses being at their highest during the first 3 months as the risk of acute rejection and allograft loss is also highest.^25^ Subsequent tapering of doses means organ recipients are then at an increased risk of community-acquired infection.^26^ The acute effects of exercise on immune function depression observed in non-transplant populations^27^ could further potentially contribute to increased infection risk. Most of the scientific evidence in support of regular exercise for transplant recipients was published in the last ten years, thus, initial advice given to the current study’s participants (~11 years prior) could justifiably have been conservative. The lack of published evidenced based practice for TxA within physiotherapy and sports therapy^28^ also appears to exist for exercise and training advice.

### Performance limitations

Over half of the TxA (53%) perceived there to be an existing limitation to their performance, the most frequent responses relating to injury or illness and lack of fitness. Although this study did not assess the specific nature of injuries, it is important to recognise that in non-transplant recipient runners, injuries such as stress fractures accounts for ~69% of all overload injuries.^29^ Chronic immunosuppressant and glucocorticoid use by transplant recipients increases the potential for osteoporosis and fractures whether involved in sport or not.^30 31^ For example, research assessing injury 8 years post-transplant reported fracture incidence as high as 46% in liver transplant recipients.^32^ Predisposing risk factors for injury include age, BMI, health, physical fitness, skill level,^33^ excessive loading, insufficient recovery and underpreparedness.^34^ A large number of TxA could therefore be at an accentuated risk of injury.

Although less than one-third of TxA considered they could train at a similar intensity as non-transplant recipient athletes, over half felt they recovered as well. The latter maybe a factor of reduced training intensity undertaken or a result of those competitors who were able to train at greater intensity also being those reporting good recovery. Interestingly, over half of the heart-recipients reported recovery was impaired with the majority of athletes reporting recovery being impeded by chronic fatigue also being heart-recipients. It is therefore possible that the heart-recipients may be more physiologically limited dependent on, among other factors, the number of medications taken and the magnitude of sympathetic nervous system re-innervation potentially affecting cardiovascular responses. Transplant athletes as a group, however, may not fully understand their own limitations. Future studies should assess the physiological responses to exercise and recovery in TxA to enable more specific exercise and training advice to be provided.

### Participation reasons

Although the physiological benefits of exercise for organ recipients have been reported,^19–21^ many other reasons exist for exercise participation including; psychological, environmental, behavioural, social and cultural factors.^35^ Within the current population four main reasons for competing were reported, two of which were indeed related to physiological aspects; to improve fitness and encourage a healthier lifestyle. The third reason reported was to compete for fun. For a group of non-transplant participants who were younger (18–25 years) but similar to the current population in terms of ethnicity (80% white) and BMI, men exercised for enjoyment more so than women who exercised for weight related reasons,^36^ a finding that has recently been observed for TxA.^37^ As the current population were Transplant Games competitors, it could be argued that both male and female participants may be more competitively motivated. However, much smaller numbers of competitors aimed to win national or international events with even fewer competing to break records, thus highlighting a predominantly enjoyment-based driver. However, the fourth reason for participation discriminates TxA from conventional athletic competitors by participating to be part of the transplant community. At transplant games events TxA are able to meet peers who have overcome similar adversity and who have received the ’gift of life’^38^ which is unique to this population. Social support from friends and family, previously identified as strong associates for physical activity,^35^ are also likely to drive TxA who have experienced significant health issues.^14 38^ and should be studied further in this population.

### Limitations

While over 200 TxA responded to the survey this was limited to English speakers from 20 nationalities, thus, the characteristics of all nations competing at the World Transplant Games are not represented. The inclusion of TxA’s from a wider selection of nations, could consolidate exercise practices and improve training recommendations. Although the predominance of white males within our TxA population is similar to previous studies^9 39^ the proportion of black, Asian and minority ethnic groups receiving organs in the UK was not reflected in our sample. Although we did not set out to assess ethnicity per se it is plausible that these recipients face additional health and cultural related challenges, for which participation at transplant events holds a lesser priority.^40 41^ Each TxA’s journey and training experience should thus be considered. Finally, the survey was only open to TxA over 18 years of age, yet a large part of the Transplant Games’ philosophy is to inspire those under 18 to increase activity and compete. An understanding of young TxA is essential to optimise their transplant journey.

## Conclusion

This study details the characteristics of TxA attending the British and World Transplant Games. With the exception of stem cell recipients, the majority of TxA manage multiple medications, often being specific to their transplant type. Many TxA’s attend the Games for the health-related benefits and fitness. Although many TxA compete for enjoyment a smaller proportion compete to win. More specific guidance post-transplant needs to be developed regarding physical activity, exercise, and reduction of injury risk. Each TxA’s journey differs with respect to transplant type and exercise experience which is likely reflected in their exercise capacity and goals.

## Data Availability

All data relevant to the study are included in the article or uploaded as online supplemental information. Not applicable.
