# Supplementary material for: Characteristics of transplant athletes competing at national and international transplant games
Source: BMJ Open Sport Exerc Med. 2022 Feb 28;8(1):e001248. doi: 10.1136/bmjsem-2021-001248 (PMC8886416; doi:10.1136/bmjsem-2021-001248)
Supplement: Supplementary data [file bmjsem-2021-001248supp002.pdf]

BMJ Publishing Group Limited (BMJ) disclaims all liability and responsibility arising from any reliance placed on this supplemental material which has been supplied by the author(s)

Supplementary Material

Table 1. Medications characteristics within the five most common transplant sub-groups related to percentage of the sub-group populations.

**Medications (One or more)**	**All**	**Kidney**	**Liver**	**Heart**	**Stem cell**	**Lung**
Number of transplant recipients	220	95	50	35	21	11
Mean number of medications $\pm$SD	5 ±3	5 ±2	4 ±3	6 ±2	2 ±2	8 ±4
Immunosuppressants	88%	95%	94%	100%	10%	91%
Statins	28%	22%	6%	80%	-	27%
Steroids	47%	65%	26%	43%	-	82%
Analgesics	1%	3%	-	-	-	-
Antibiotics / antiviral / antifungal	20%	9%	12%	23%	34%	90%
Anti-hypertensives	47%	58%	28%	69%	10%	36%
Anti-inflammatories	4%	1%	16%	-	-	-
Antacid inhibitors	20%	24%	14%	23%	5%	18%
Anti-diabetic	10%	9%	8%	3%	-	63%
Anti-platelets	22%	20%	26%	37%	5%	-
Anti-coagulants	2%	1%	2%	6%	-	9%
Anti-epileptic	1%	2%	-	-	-	-
Anti-depressant / anxiolytics	7%	4%	4%	9%	14%	9%
Anti-retroviral	1%	-	4%	-	-	-
Antihistamine	2%	-	2%	3%	-	9%
Antiparkinsonian	0.5%	1%	-	-	-	-
Respiratory medication	1%	1%	-	-	-	9%
Hormone therapy	11%	9%	6%	20%	19%	-

Retanoid	1%	1%	2%	-	-	-
Triptants	1%	-	4%	3%	-	-
Gout treatment	8%	8%	2%	20%	-	9%
Gastrointestinal medication	13%	8%	24%	6%	5%	27%
Diuretics	2%	-	25	3%	-	-
Prostate cancer medication	0.5	1%	-	-	-	-
Vitamin /minerals	36%	33%	38%	37%	10%	63%
